# Letter from Dr. Lowry; His Case of Extra-Uterine Pregnancy

**Published:** 1890-02

**Authors:** S. T. Lowry

**Affiliations:** San Antonio, Texas


### Correspondence.

### LETTER FROM DR. LOWRY.—HIS CASE OF EXTRA-UTERINE PREGNANCY.

SAN ANTONIO, TEXAS, February 10, 1890.

*Editor Daniel's Texas Medical Journal:*

Some weeks ago I wrote for the Courier-Record of Medicine the history of a case which I termed "Extra-Uterine Pregnancy." The criticisms through your journal have been such as to warrant me in claiming at your hands a brief hearing in reply.

Those who write for medical journals may expect to have their productions carefully scanned by their professional brethren, and just criticisms, delivered in a fair and courteous manner, should give no offense. In the vast unlimited domain of general medicine and surgery there is so much to know, and the best of us know so little that we all feel our weakness and ignorance when we contemplate the subject, and are not surprised nor offended, when our mistakes are pointed out to us in a proper spirit. But when we are held up to ridicule, and made to appear ignorant of things that any tyro in medicine ought to know, we feel that the legitimate fruits of just criticism are lost, and that the channels of current medical literature are being diverted from their proper course.

Let me briefly outline the history of this case: I was called to see a woman seven months pregnant, after the membranes had ruptured and a large quantity of water had flowed away. I found on palpation of the abdomen the outlines of a uterus about the size of a child's head just above the symphysis pubis and in the median line. To the right of the uterus I could plainly make out the irregular outlines of a larger body, which, I soon found, was connected with the uterus by an attachment seemingly about two inches in diameter, with just space enough between the two bodies to allow the tips of the fingers to be pressed down and definitely outline the connection. Digital examination revealed an os patulous, but not sufficiently so to allow the free introduction of the finger. I explored the uterine cavity, however, with a large blunt sound, and found it perfectly empty. Deflecting the sound to the right side of the uterus in the region of the Fallopian opening, it readily passed through an outlet into the cavity which contained the irregularly shaped body before mentioned, which could be easily felt and which afterwards proved to be a fœtus. In an experience with many hundreds of obstetrical cases, I am free to confess that this was a novel situation to me. What was the actual condition of things, and what was to be done? Would it be possible to dilate the os, pass the hand into the uterus and then through this second opening and remove the fœtus *per vias naturalis?* Or, if this was impossible, would laparotomy become necessary, and when? The woman resting in comparative comfort, there was no occasion for hasty action.

In about two days the fœtal head could be felt in the cavity of the uterus; expulsive pains came on and a very small child was delivered without instrumental interference. The placenta was retained. I introduced my hand into the uterine cavity and again found it empty; I followed with my hand the cord through an opening *in the region of* the tubal attachment into the cavity where the fœtus had first been found. Here I also found the placenta adhered to the inner surface of this sac or cavity, and succeeded in detaching it. Hemorrhage was not profuse, and the woman made a good recovery. Further examination to confirm some points in the diagnosis was not allowed. Now, these are the facts in the case. Each reader can draw his own conclusions. I am well aware, that Fallopian tubes do not often contain full term fœtus, although so renowned a writer as Thomas says, "Extra-uterine pregnancy of any variety may reach full term, no injury resulting to mother or child." I am also aware that the hand cannot be thrust through a Fallopian opening and tear a placenta from the abdominal viscera with impunity. Nor do I contend that this was a case of tubal, abdominal, intestitial, or ovarian pregnancy. But I do contend that it was an unique and peculiar case of ectopic gestation—a case of *extra*-uterine pregnancy, in the literal meaning of that term; and while the fœtus was not contained in the Fallopian tube, perhaps, as that tube is usually found developed in such pregnancies, still there was some development, growth, prolongation, horn, or attachment just where the tube is found; that this growth was not the uterus, but only connected to it by a narrow neck; that it must also have been connected with the Fallopian tube, else impregnation could not have taken place in it, and it must have been composed of muscular tissue, as the fœtus was forced out of it.

Now, I have not sought to make a wonderful case out of this, but simply to state the facts as I understood them, and arrive at a rational conclusion as to the actual condition. While several have cried out "mistaken diagnosis," when I have really made no diagnosis at all; still of the many criticisms that I have read in public print, and private letters received, only two have offered any explanation of the case. One of these, which was published in your journal, explained the matter by saying that I had mistaken a natural pregnancy with hour-glass contraction of the uterus for tubal pregnancy. Now, in reply to this, laying aside many things that might be said, I submit that a uterus in the throes of a three days hour-glass contraction, would be more wonderful, than a tubal pregnancy at full time. Another explanation, submitted in a private letter, with drawing attached, by a prominent professor and surgeon, was, that the pregnancy took place in a horn of the uterus which projected from its body at the point of tubal attachment on the right side. That as the fœtus grew, the horn enlarged, except near the uterus, which gave the appearance of two bodies connected by a constricted attachment. This seems a very rational explanation.

S. T. LOWRY.

